# Primary Hepatic Neuroendocrine Tumor: A Case Report and Literature Review

**DOI:** 10.7759/cureus.22370

**Published:** 2022-02-18

**Authors:** Ahmad Elayan, Hamzeh Batah, Moath Badawi, Ahmad Saadeh, Sufian Abdel Hafez

**Affiliations:** 1 Radiology, Jordan Ministry of Health, Amman, JOR; 2 Pathology, Jordan Ministry of Health, Amman, JOR; 3 General Practice, Faculty of Medicine, The University of Jordan, Amman, JOR; 4 Internal Medicine, Faculty of Medicine, The University of Jordan, Amman, JOR

**Keywords:** surgical case report, immunohistochemistry staining, zollinger-ellison syndrome, solitary hepatic mass, primary neuroendocrine tumor

## Abstract

Primary hepatic neuroendocrine tumors (PHNETs) are an utterly rare subtype of neuroendocrine tumors (NETs) that arise from cells of the neuroendocrine system. Due to the rarity and lack of distinctive radiological features, diagnosis and management of this tumor are challenging. Herein, we report a case of PHNET in a 19-year-old previously healthy female patient whose diagnosis was confirmed by histopathology and immunohistochemistry. This case emphasizes the importance of considering PHNETs in the differential diagnosis of a hepatic mass, management of patients with this disease, and post-operative follow-up.

## Introduction

Neuroendocrine tumors (NETs) are rare neoplastic growths that arise from cells of the neuroendocrine system. They predominantly occur in the gastrointestinal tract and the respiratory tract [1]. The liver is mostly involved by metastases of NETs whereas primary hepatic neuroendocrine tumors (PHNETs) represent an utterly rare subtype of NETs approaching only 200 cases in the literature without sex propensity, and with a mean age at diagnosis of about 47-50 years with only a few reported cases where the patients are younger than 40 years [2-4]. PHNETs are slowly growing tumors which leads to their presentation at a late stage [3]. They are considered challenging to diagnose owing to the fact that they lack distinct radiological features which leads to misdiagnosing them with other liver lesions [1,3,5]. Considering that, definitive diagnosis can be achieved by histopathology and immunohistochemistry [2,6]. Herein, we report a case of PHNET in a 19-year-old previously healthy female patient whose diagnosis was confirmed by histopathology and immunohistochemistry.

## Case presentation

A 19-year-old previously healthy pregnant (seventh week) lady presented to our emergency department (ED) complaining of vomiting, melena, abdominal pain, shortness of breath, and generalized weakness. She had episodic diarrhea and mild right upper quadrant pain during the two weeks, prior to her presentation to ED. She was not taking any regular medications except for prenatal vitamins. On physical examination, she had blood pressure of 101/50 mmHg, heart rate of 145 beats per minute (bpm), respiratory rate of 34 breaths per minute, temperature of 37.1°C, with signs of dehydration and right upper abdominal tenderness and fullness. Abdominal ultrasound showed a large heterogeneous mainly hypoechoic lesion in the right liver lobe, with a maximum dimension of about 10 cm and pelvic ultrasound revealed an absence of fetal heart activity while blood work showed a hemoglobin (Hb) level of 4 g/dL. After stabilizing the patient and performing blood transfusion (four units of packed RBCs and fresh frozen plasma), she underwent an upper GI endoscopy in which gastric and duodenal inflammation was found with multiple duodenal ulcers which were clear of malignant changes on biopsies. Additionally, no masses and no source of active bleeding were evident. A subsequent lower GI endoscopy was unremarkable. It is of note that the patient had no prior history or laboratory evidence of *Helicobacter pylori* infection.

**Figure 1:**
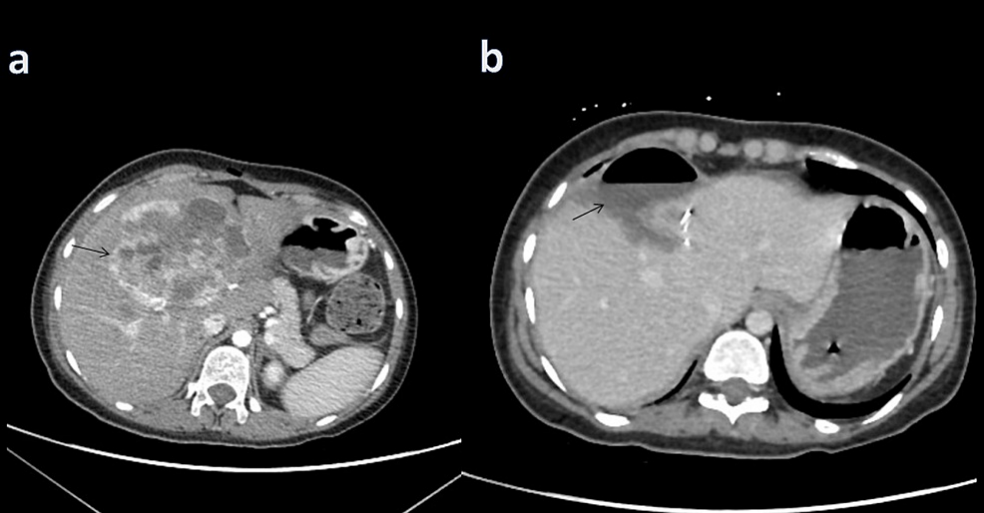
Abdominal CT scan showing (a) a large heterogeneous hypodense irregular lesion in the right liver lobe with peripheral enhancement on the arterial phase and (b) post wedge resection of the liver. Post-operative changes are noted and a fluid collection is seen anterior to the liver.

Chest x-ray did not reveal any pertinent changes while triphasic CT scan showed a large heterogeneous hypodense irregular lesion in the right lobe of the liver with peripheral enhancement on the hepatic arterial phase with a measurement of 8.7x10^9^ cm (Figure 1, panel a). There was interrupted enhancement on the venous phase, while on the delayed phase, the lesion was still heterogeneous with central irregular hypodensity and isodensity on the periphery. The rest of liver parenchyma was homogenous with no signs of cirrhosis. Pan-CT showed no distant sites of metastasis and positron emission tomography (PET) scan was not available. Serum gastrin level was 850 pg/mL (normally <100 pg/mL) which along with the clinical and endoscopic findings is consistent with Zollinger-Ellison syndrome (ZES) caused by gastrin-secreting neoplastic cells. Other tumor markers (alpha-fetoprotein {AFP} and carcinoembryonic antigen {CEA}) were normal.

**Figure 2:**
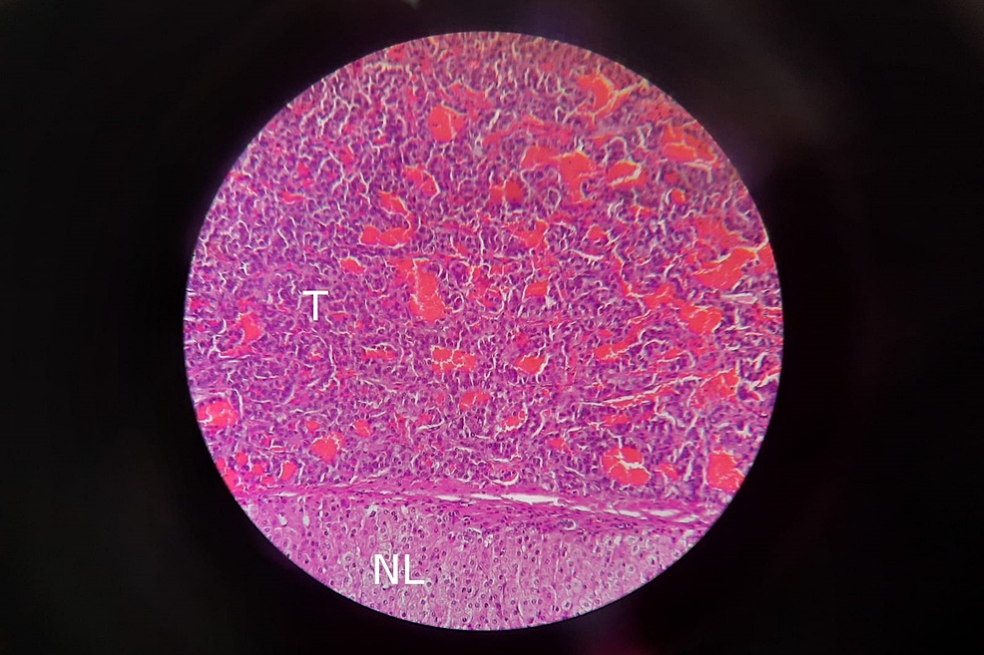
Histopathology slide was taken from the liver mass showing features of neuroendocrine tumor on H&E stain. The part labeled T shows tumor cells, while the part labeled NL represents normal liver tissue.

Following that, the patient was scheduled for surgery and a liver wedge resection was performed during which the mass was removed with a safety margin close to the hepatic artery and portal vein (Figure 1, panel b). Histopathology of the resected lesion revealed areas of necrosis and features suggestive of a neuroendocrine tumor (Figure 2).

**Figure 3:**
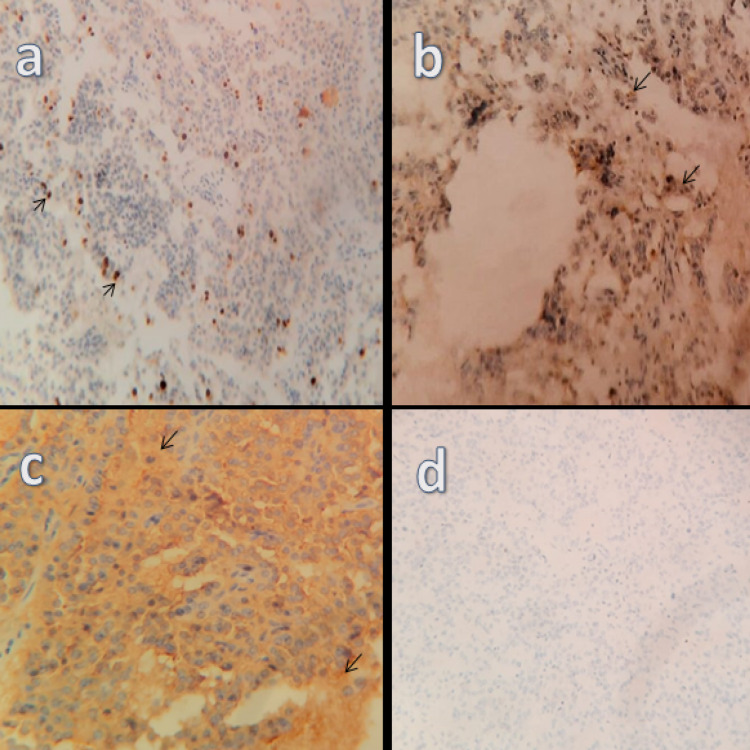
Immunohistochemistry showing tumor reactivity to (a) Ki-67, (b) synaptophysin, (c) CD56, and (d) an absence of reactivity for CDX2. CD56: cluster of differentiation 56; CDX2: caudal type homeobox 2

Regional lymph nodes demonstrated reactive hyperplasia without neoplastic changes. On immunohistochemistry, the lesion was positive for cluster of differentiation 56 (CD56), cytokeratin (CK)8/18, epithelial membrane antigen (EMA), and synaptophysin (Figure 3, panels a-d). The Ki-67 index was 12% indicating a grade 2 tumor. On the other hand, the tissue tested negative for AFP which is a known marker for hepatocellular carcinoma, and caudal type homeobox 2 (CDX2), thyroid transcription factor 1 (TTF1), and pancreatic and duodenal homeobox 1 (PDX1) which aided in ruling out an extra-hepatic origin for the cells. It is of note that gastrin stain was not available.

The patient recovered well after the procedure without any complications and her gastrin returned to normal levels. Since the tumor was isolated and resectable, other modalities of treatment were not pursued post-operatively. On subsequent follow-ups over the next year, she remained asymptomatic with no further complaints or evidence of recurrence.

## Discussion

Liver neoplasms are rare and potentially life-threatening diseases. NETs are one of the rarest subtypes and constitute less than 1% of primary liver tumors resected [4]. NETs are neoplastic growths of neuroendocrine cells that mostly occur in the GI and respiratory tracts [1]. The involvement of the liver is most commonly due to metastasis of NETs [5]. PHNETs are a rare subtype of NETs, they comprise less than 0.3% of NETs with vagueness in their origin [4,7]. Some argue that they are caused by ectopic pancreatic tissue, others argue that they originate from progenitor cells in the intrahepatic bile ducts, yet no consensus has been established due to their rarity [4,8]. PHNETs are rarely present in patients under the age of 40 years, and previous studies did not find a specific sex predilection for the condition [4]. Our patient was diagnosed with PHNET at the age of 19 years. To the best of our knowledge, this is the first case of a PHNET published from Jordan.

PHNETs can be classified in terms of functionality into functional PHNETs and non-functional PHNETs. They are distinct from other NETs since they mostly present as non-functional tumors [9]. Considering that, they grow asymptomatically and are usually present at late stages [10]. Patients may experience abdominal pain, abdominal distension, or jaundice attributed to the mass effect of the tumor [9-11]. Functional PHNETs may cause symptoms of ZES, Cushing's syndrome, and carcinoid syndrome although they are uncommon in patients with PHNETs [4,10]. Our patient presented with abdominal pain and recurrent diarrhea with an endoscopic finding of multiple duodenal ulcers and a high gastrin level, all of which point towards ZES. Gastrin levels returned to normal post-operatively which further supports our impression. ZES is a common presentation of gastrinomas which are gastrin-producing neuroendocrine tumors. Primary hepatic gastrinoma has been described in only 35 cases as reported in a recent literature review [12]. Additionally, she was found to have a miscarriage of her seventh week pregnancy during her ED presentation. To the best of our knowledge, no previous studies have linked the pathogenesis or prognosis of PHNETs with pregnancy, hence we encourage future researchers to explore any possible associations.

As most PHNETs are asymptomatic, early diagnosis is a real struggle [10]. The diagnostic approach is a continuous process pre- and post-operatively [1]. Imaging modalities such as ultrasound, CT scan, and, MRI have a cornerstone role in diagnosing liver masses in general. PHNETs mostly have generous blood supply but lack distinctive radiological features from other liver tumors and are commonly misdiagnosed as hepatocellular carcinoma [9,13]. Serum tumor markers such as alpha-fetoprotein (AFP) and carcinoembryonic antigen (CEA) have no practical diagnostic value in diagnosing PHNETs [13]. In our case, a hepatic mass was unexpectedly picked up by abdominal ultrasound and a CT scan with contrast was done showing a large heterogeneous hypodense irregular lesion in the right liver lobe that showed peripheral enhancement on the arterial phase.

Histopathology examination is one of the best ways to diagnose PHNETs with high accuracy [14]. A biopsy can be obtained by a fine needle or post-surgically after resecting the mass. However, fine needle biopsy has not proved to be accurate enough [6]. Usually, biopsies are stained with hematoxylin and eosin dye which helps in tumor classification. However, other stains can be used to elevate the accuracy of the diagnosis such as Masson’s stain [13]. Immunohistochemistry is the most accurate method to reach a definitive diagnosis of PHNETs [6]. NETs are usually positive for synaptophysin, chromogranin, CD56, and neuron-specific enolase. In addition to that, Ki-67 is used to assess the ratio of positive tumor nuclei to others [1]. Other immunohistochemistry stains have shown the ability to detect the origin of the tumor such as CDX2 which points toward gastroenteropancreatic origin, TTF-1 with tumors of a thoracic origin, and others that are used to point out the primary NETs [4]. In our case, the biopsy was taken post-operatively and stained with hematoxylin and eosin dye. Immunohistochemistry was positive for CK8/18, EMA, CD56, synaptophysin, and the Ki-67 was (12%) while it was negative for CDX2, TTF-1, PDX1. Unfortunately, gastrin stain was not available for immunohistochemical confirmation of a gastrinoma.

Treatment of PHNETs can be categorized as surgical and medical with the surgical treatment being the most effective option so far as it showed a 74% five-year survival rate with recurrence rate of nearly 18% [15,16]. Resection is determined by the size and location of the tumor with tumors of multiple foci that involve both liver lobes being treated with other modalities such as selective hepatic artery embolization, radiofrequency ablation, and hepatectomy with liver transplant [17]. Medical treatments such as transcatheter arterial chemoembolization (TACE), systemic chemotherapy, local ablation, and somatostatin analogs have failed to prove a long-term survival benefit. TACE showed a good initial response in the short term only [15,17]. In addition to that, TACE can reduce tumor size which would be beneficial prior to surgical resection [15]. Due to the rarity of the disease, recommendations or a consensus on a treatment plan is difficult [17]. Our patient underwent liver wedge resection as the tumor was solitary, she was followed up for one year without any complaints and no symptoms have been reported so far.

## Conclusions

Primary hepatic neuroendocrine tumors (PHNETs) constitute a unique entity in their pathogenesis, prevalence, and eluding medical and radiological presentation. Zollinger-Ellison syndrome (ZES) caused by a primary hepatic gastrinoma is an example in which it can present. Thus, it is challenging to diagnose this disease, manage it, and confirm a primary origin with an absence of metastases. Surgical management is currently the best approach to pursue with subsequent histopathological and immunohistochemical confirmation and regular follow-ups. Raising awareness of its possibility in atypical patient age groups and its various presentations will contribute to surgeons’ ability to detect and manage it appropriately.
